# Pancreatic enzyme replacement therapy in subjects with exocrine pancreatic insufficiency and diabetes mellitus: a real-life, case–control study

**DOI:** 10.1186/s13098-024-01265-4

**Published:** 2024-02-09

**Authors:** Laure Alexandre-Heymann, Fetta Yaker, Pierre Bel Lassen, Danièle Dubois-Laforgue, Etienne Larger

**Affiliations:** 1 Service de Diabétologie, AP-HP, Hôpital Cochin, 123 boulevard de Port-Royal, 75014 Paris, France; 2 Institut Cochin, INSERM, CNRS, Université Paris Cité, Paris, France; 3 Sorbonne Université, INSERM, Nutrition and Obesities: Systemic approaches (NutriOmics), Paris, France; 4 Service de Nutrition, AP-HP, Hôpital Pitié-Salpétrière, Paris, France

**Keywords:** Fecal elastase, Pancreatic enzyme replacement therapy, Exocrine pancreatic insufficiency, Diabetes

## Abstract

**Background:**

Exocrine pancreatic insufficiency (EPI) can be associated with all types of diabetes. Pancreatic enzyme replacement therapy (PERT) has short and long-term benefits in subjects with EPI, but its effects on diabetes control are uncertain. We aimed to study the effects of PERT initiation on glycemic control in subjects with diabetes and EPI from any cause.

**Methods:**

In this retrospective study, we compared subjects with EPI and diabetes who were prescribed PERT with subjects with diabetes who had a fecal elastase-1 concentration dosage, but did not receive PERT. The primary outcome was the effect of PERT on hypoglycemia frequency and severity. The secondary outcomes were the effects of PERT on gastro-intestinal disorders, HbA_1c_ and body mass index (BMI).

**Results:**

48 subjects were included in each group. Overall, PERT did not have any significant effect on hypoglycemia frequency or severity, but hypoglycemia frequency tended to decrease in subjects with chronic pancreatitis. While 19% of subjects experienced mild hyperglycemia after PERT initiation, we did not report any keto-acidosis or any other severe adverse event. Gastro-intestinal disorders improved in 80% of subjects treated with PERT, versus in 20% of control subjects (*p* = 0.02). Gastro-intestinal disorders improved in 87% of subjects with recommended dosage of PERT, versus in 50% of subjects with underdosage (NS). HbA_1c_ and BMI evolution did not differ between the groups.

**Conclusions:**

PERT initiation is safe in subjects with diabetes and EPI. It does not globally decrease hypoglycemia severity of frequency, but is associated with a decrease in gastro-intestinal disorders.

*Trial registration* Retrospectively registered. The database was registered with the Commission Nationale Informatique et Libertés (CNIL), registration number: 2203351v0. The study was approved by the local ethics committee CLEP, registration number: AAA-2023-09047

## Background

Exocrine pancreatic insufficiency (EPI) is a hallmark of diabetes mellitus secondary to pancreatic diseases, also known as pancreatogenic diabetes. This type of diabetes can affect subjects with acute pancreatitis [1], chronic pancreatitis (CP), pancreatic cancer, or cystic fibrosis. Pancreatogenic diabetes is often misdiagnosed with either type 1 (T1D) or type 2 diabetes (T2D) and could represent up to 10% of the causes of diabetes, at least among hospitalized subjects [2]. However, clinically relevant EPI can also be observed in 11 to 30% of subjects with T1D [3, 4], 8% of subjects with T2D [5], and in subjects with several types of Maturity Onset Diabetes of the Young (MODY) [6].

It is important to screen for EPI in subjects with diabetes and suggestive symptoms, e.g. abdominal pain, weight loss, maldigestion, or frequent episodes of hypoglycemia. Indeed, EPI associates with complications such as weight loss, diarrhea and steatorrhea, malnutrition [7], osteoporosis, and increased cardiovascular risk [8], and many studies have shown that treating EPI with pancreatic enzyme replacement therapy (PERT) has both short- and long-term benefits [9]. On the short- to medium-term, PERT improves fat and nitrogen absorption [10], induces weight gain [11], and decreases gastro-intestinal symptoms frequency and severity [12, 13]. On the long-term, PERT improves quality of life [14, 15], decreases the risk of bone fractures [16], and improves survival in subjects with CP or with pancreatic cancer [17, 18].

Subjects with pancreatogenic diabetes may develop “brittle diabetes” with rapid glycemic shifts and risk of severe hypoglycemia and hyperglycemia [19–21]. However, only a few studies have been conducted on the effects of PERT on glycemic control: PERT has been shown to improve glucagon-like peptide-1 (GLP1) and glucose-dependent insulinotropic polypeptide (GIP) secretion [22] and is thus expected to affect gastric emptying and postprandial blood glucose concentration. Indeed, in the context of cystic fibrosis, studies showed that PERT normalized GLP-1 secretion, slowed gastric emptying, and decreased postprandial blood glucose without completely normalizing it [23, 24]. In another study performed on subjects with fibrocalculous pancreatitis, PERT was associated with lower HbA_1c_ and postprandial plasma glucose [25]. However, studies performed on small numbers of subjects with CP or T2D did not show any effect of PERT on post-prandial blood glucose, C-peptide secretion or on gastric emptying [26–28]. Conversely, PERT could also worsen glycemic control by increasing nutrients absorption. In one study performed on 18 subjects with CP and diabetes, one subject had major difficulties to attain acceptable glycemic control and one developed ketoacidosis after PERT initiation, while two subjects experienced severe hypoglycemia after ceasing PERT [29]. However, these complications were not confirmed in other studies [10, 26]. A randomized, versus placebo study in subjects with insulin-treated diabetes mellitus and EPI showed that PERT could be used safely, and reduced the frequency of mild and moderate hypoglycemia [30].

Therefore, we aimed to study the real-life effects of PERT initiation on glycemic control in subjects with diabetes and EPI from any cause. In this study, we compared subjects with EPI and diabetes who were prescribed PERT with subjects with diabetes who had a relatively low fecal elastase-1 concentration (FEC) dosage, but did not receive PERT. The primary outcome was the effect of PERT on hypoglycemia frequency and severity. The secondary outcomes were the effects of PERT on gastro-intestinal disorders, HbA_1c_, body mass index (BMI), and on overall diabetes management.

## Methods

### Subjects

We conducted a retrospective study comparing participants with diabetes with or without PERT initiation. It was conducted at a tertiary referral center (the Diabetology Department of Cochin Hospital, Paris).

All subjects followed in our Diabetology Department who had PERT initiation between September 2013 and September 2022, and who were diagnosed with diabetes at least 3 months before PERT initiation, and who had severe EPI as defined by a FEC < 100 µg/g of stool, and who had at least one consultation in the department within the 2 years following PERT initiation were included in the PERT group. Subjects were defined has having PERT initiation if they received a prescription of pancreatin gastro-resistant capsules for the first time. In France, pancreatin is sold under 2 brand names: CREON® (Viatris Santé, France) or EUROBIOL® (Mayoly Spindler, France).

For the control group, we selected subjects followed in our Diabetology Department who did not receive PERT, matched on age and gender with the subjects from the PERT group (1:1). We selected subjects in whom a FEC dosage was performed between January 2016 and September 2022 with a relatively low result (< 300 µg/g), in order to get clinically similar subjects to the PERT group. To be included, they also had to be diagnosed with diabetes at least 3 months before FEC dosage and to have at least one consultation in the department within the 2 years following FEC dosage.

### Materials and methods

We extracted all biological, radiological and clinical data from the subjects’ electronic medical records.

T_0_ (index date) was defined as the date of initiation of PERT for the subjects from the PERT group, and as the date of FEC dosage for the subjects from the control group.

#### Hypoglycemia

Frequent hypoglycemia was defined as the occurrence of at least 4 episodes of documented symptomatic hypoglycemia per week. Severe hypoglycemia was defined as hypoglycemia requiring the assistance of another person to administer glucose or sugar. Subjects were classified as having recent severe hypoglycemia if they experienced at least one episode of severe hypoglycemia within the last 2 years before T_0_. Subjects were classified as having “relevant hypoglycemia” if they experienced frequent documented symptomatic hypoglycemia and/or experienced recent severe hypoglycemia. Improvement of hypoglycemia was deemed present if, during their follow-up visits (between 3 months and 2 years after T_0_), the subjects reported that the frequency of relevant hypoglycemia decreased.

#### Gastro-intestinal disorders

Subjects were classified as having gastro-intestinal disorders if they reported experiencing diarrhea, steatorrhea, nausea, or abdominal pain at least once a week. Improvement of gastro-intestinal disorders was deemed present if, during their follow-up visits (between 3 months and 2 years after T_0_), subjects reported that the frequency of their usual digestive symptoms decreased.

#### Recommended dosage of PERT

The European guidelines for the therapy of chronic CP recommend treating subjects with CP and EPI with enzyme replacement therapy (pancrelipase/pancreatin) of 40,000–80,000 units of lipase during main meals and half dose during snack meals [31, 32]. Subjects from the PERT group were classified into the recommended dosage (RD) subgroup if they were prescribed the appropriate dosage and in the underdosage (UD) subgroup if they were prescribed lower dosages.

#### HbA_1c_

To study the effect of PERT on HbA_1c_, we defined baseline HbA_1c_ as the mean HbA_1c_ of each subject over the year prior to T_0_, and follow-up HbA_1c_ as the mean HbA_1c_ over the year following T_0_. Intra-subject variability was defined as follows: intra-subject variability of HbA_1c_ = follow-up HbA_1c_ of subject – baseline HbA_1c_ of said subject.

#### Body mass index

To study the effect of PERT on BMI, we defined baseline BMI as the mean BMI of each subject over the 2 years prior to T_0_, and the follow-up BMI as the mean BMI over the 2 years following T_0_. Intra-subject variability was defined as follows: intra-subject variability of BMI = follow-up BMI of subject – baseline BMI of said subject.

#### Dosages

Vitamin A deficiency was defined as a vitamin A serum dosage < 1.5 µmol/L. Vitamin E deficiency was defined as a vitamin E serum dosage < 21 µmol/L.

### Statistical analysis

Statistics were performed using Graphpad Prism 9 (GraphPad Software, LLC).

Data are presented as mean and standard deviation (SD) for normally distributed variables, and as median and interquartile range (IQR) for variables with a non-normal distribution. For continuous variables, when the PERT and control groups were compared, we used unpaired t-test for normally distributed variables and Mann-Whitney test for variables with a non-normal distribution. When we studied intra-subject variability before and after T_0_, we used paired t-test for normally distributed variables and Wilcoxon test for variables with a non-normal distribution. For categorical variables, we used Fisher’s exact test when we compared 2 groups and Chi-square when we compared more than 2 groups. A p-value < 0.05 was considered statistically significant.

When data was missing, we reported the number of analysable participants in the corresponding table or figure.

Among the PERT group, we performed a subgroup analysis to compare subjects who were prescribed the recommended dosage of PERT with the ones who were not (RD and UD subgroups).

## Results

Forty-eight subjects were included in each group, 73% of the subjects were men (Table 1). Subjects treated with PERT presented more often with pancreatogenic diabetes than control subjects, who presented more often with T1D or T2D. Subjects treated with PERT had a lower BMI than control subjects. About 75% of subjects were treated with insulin in both groups. FEC was lower, and gastro-intestinal disorders, pancreas anomalies on CT, and vitamin A deficiency were more frequent in subjects treated with PERT than in control subjects. However, rates of relevant hypoglycemia were similar between groups.

**Table 1:** Characteristics of the subjects

	Subjects treated with PERT (*n* = 48)	Control group (*n* = 48)	p
Subjects’ characteristics			
Age: years (mean, SD)	57 (14,8)	56 (14,3)	0.97
Male: n (%)	35 (73)	35 (73)	
Tobacco use			0.38
Never: n (%)	17 (35)	23 (48)	
Active user: n (%)	16 (33)	15 (31)	
Former user: n (%)	15 (31)	10 (21)	
Alcohol consumption			0.89
Never: n (%)	21 (44)	23 (48)	
Occasional drinking: n (%)	13 (27)	10 (21)	
Regular alcohol use: n (%)	5 (10)	6 (13)	
Excessive drinking, current or stopped: n (%)	9 (19)	8 (17)	
BMI at T_0_: kg/m^2^(median, IQR*)*	22.6 (19.9–26.7)	27.6 (22.5–29,6)	0.0022
Loss of weight at T_0_ as compared to maximum weight ever: kg (median, IQR)	9.5 (4–16.,3)	8.5 (3–15)	0.63
Diabetes			
Diabetes duration: years (median, IQR)	12.1 (4.1–27.2)	14.9 (7.2–29.1)	0.59
Diabetes type			< 0.0001
Type 1 diabetes: n (%)	9 (19)	18 (38)	
Type 2 diabetes: n (%)	8 (17)	21 (44)	
Pancreatogenic diabetes: n (%)	18 (37)	0	
Undetermined: n (%)	9 (19)	8 (17)	
Other types (post-transplantation, post-PLD1 treatment, MODY): n (%)	4 (8)	1 (2)	
Diabetes treatment at T_0_			
Insulin: n (%)	37 (77)	36 (75)	> 0.99
Metformin: n (%)	14 (29)	21 (44)	0.2
Sulfonylurea or repaglinide: n (%)	13 (27)	13 (27)	> 0.99
GLP1 agonist: n (%)	2 (4)	7 (15)	0.16
DPP4 inhibitor: n (%)	7 (15)	4 (8)	0.5
HbA_1c_ at T_0_: % (median IQR)	8.7 (6.9–9.9)	8.4 (7.4–9.6)	0.94
HbA1c at T_0_: mmol/mol (median, IQR)	72 (52–85)	68 (57–81)	
Retinopathy: n (%)	28 (58)	27 (56)	0.99
Neuropathy: n (%)	25 (52)	22 (46)	0.68
Nephropathy: n (%)	17 (35)	21 (44)	0.53
Ischemic cardiopathy: n (%)	7 (15)	11 (23)	0.43
Fasting C-peptide: nmol/L (median, IQR)	0.19 (0.04–0.36)	0.12 (0-0.33)	0.32
Exocrine pancreas			
Fecal elastase-1 concentration: µg/g (median, IQR)	26.5 (0–72)	129 (102-192.8)	< 0.0001
Anomalies on pancreas CT^†^			
Pancreas atrophy: n (%)	28 (65)	8 (23)	0.0002
Pancreas calcifications: n (%)	19 (43)	5 (14)	0.0068
Vitamin A deficiency: n (%)^‡^	8 (24)	0	0.007
Vitamin E deficiency: n (%)^§^	9 (28)	2 (7)	0.051
Exocrine pancreatic insufficiency associated symptoms			
More than 4 episodes of documented symptomatic hypoglycemia per week: n (%)	14 (29)	12 (25)	0.8
Recent history of severe hypoglycemia: n (%)	13 (27)	8 (17)	0.33
Relevant hypoglycemia (more than 4 events per week and/or recent severe hypoglycemia): n (%)	15 (31)	15 (31)	1
Gastrointestinal disorders: *n (%)*	21 (44)	10 (21)	0.02

PERT: Pancreatic Enzyme Replacement Therapy; SD: standard deviation; BMI: body mass index; IQR: interquartile range; CT: computed tomography

^†^Available in 44 subjects with PERT and 35 control subjects; ^‡^available in 33 subjects with PERT and 26 control subjects; ^§^available in 32 subjects with PERT and 27 control subjects

### Comparison between subjects with and without PERT

#### Relevant hypoglycemia

**Fig. 1:**
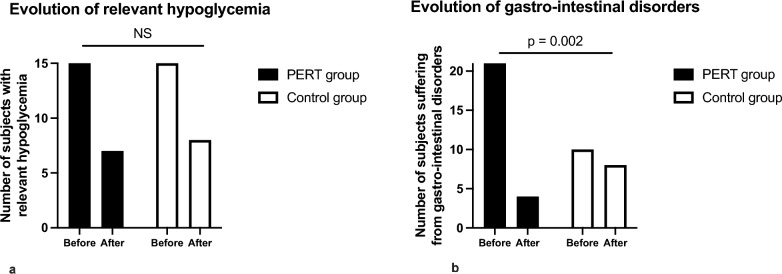
Evolution of relevant hypoglycemia and of gastro-intestinal disorders over the study period. Black: PERT group White: Control group. **a** Evolution of relevant hypoglycemia in subjects treated with PERT and in control subjects, before and after T_0_. Relevant hypoglycemia: at least 4 documented symptomatic hypoglycemia events per week and/or at least one episode of severe hypoglycemia within the last 2 years before T_0_. **b** Evolution of gastro-intestinal disorders before and after T_0_ in subjects treated with PERT and in control subjects

In both groups, 15 subjects (31%: 4 subjects with T1D, 1 with T2D, 5 with pancreatogenic diabetes, 1 with MODY-HNF1B and 4 undetermined in the PERT group; 10 subjects with T1D, 2 with T2D, 1 with post-transplantation diabetes and 2 undetermined in the control group) presented with relevant hypoglycemia. In both groups, relevant hypoglycemia was only noted in subjects treated with insulin, even though sulfonylureas were often prescribed in subjects with oral diabetes medication only. 41% of the subjects treated with PERT and insulin and 42% of the control subjects treated with insulin had relevant hypoglycemia. Hypoglycemia symptoms improved in 8 subjects after initiation of PERT, but also in 7 control subjects during the follow-up period (Fig. 1a). Of note, hypoglycemia symptoms improved in 4 out of the 5 subjects with pancreatogenic diabetes after PERT initiation (80%), versus in only 40% of subjects with other types of diabetes (NS).

#### Gastro-intestinal disorders

44% of the subjects treated with PERT (6 subjects with T1D, 6 with pancreatogenic diabetes, one with MODY-HNF1B, one with post-PDL1 treatment diabetes and 9 undetermined) and 21% of the control subjects (5 subjects with T1D, 3 with T2D, one with post-transplantation diabetes and 1 undetermined) presented with gastro-intestinal disorders. These symptoms improved in 80% of subjects after initiation of PERT, versus in 20% of control subjects during the follow-up period (*p* = 0.002) (Fig. 1b). Gastro-intestinal disorders improved in all patients with T1D and pancreatogenic diabetes treated with PERT, and did not improve in 4 out of the 9 subjects with undetermined type of diabetes. In the control group, gastro-intestinal disorders improved in one subject with T2D and in one with post-transplantation diabetes.

#### HbA_1c_

**Fig. 2:**
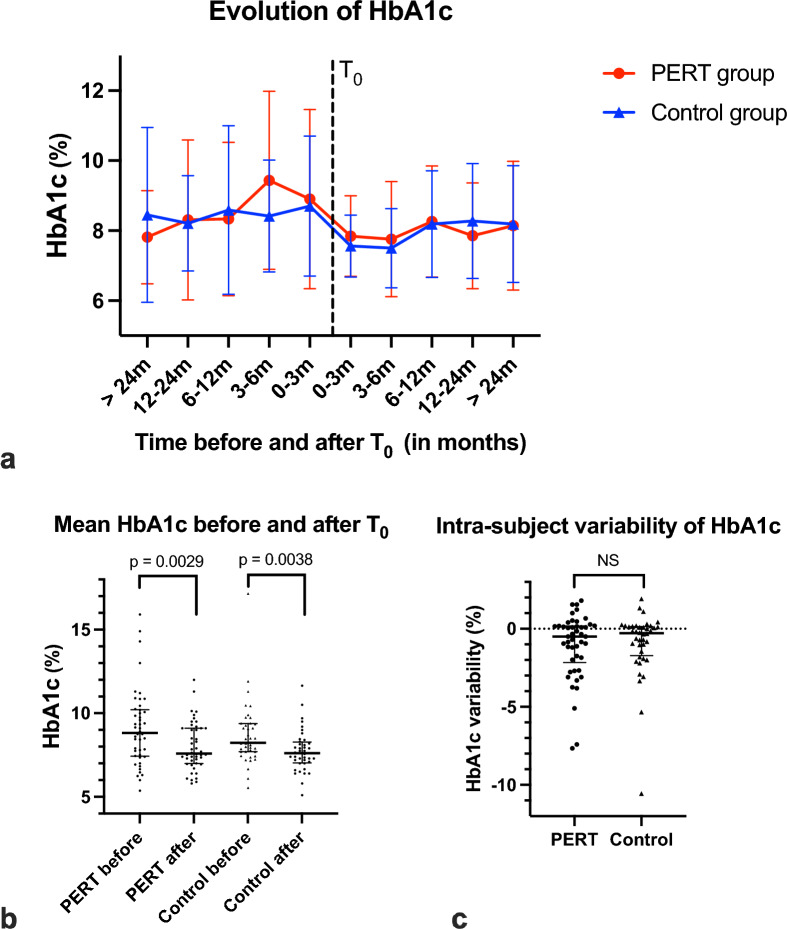
Evolution of HbA_1c_ over the study period. **a** HbA_1c_ (in %) at different time points before and after index date (T_0_). Red circles: PERT group. Blue triangles: control group. **b** Mean HbA_1c_ (in %) over the year prior and the year following T_0_ in the PERT and control groups. Each dot or triangle represents a subject (*n* = 46 in the PERT group, 40 in the control group). **c** Intra-subject variability of HbA_1c_ in the PERT and control groups. Intra-subject variability of HbA_1c_ = mean HbA_1C_ of subject over the year following T_0_ – mean HbA_1c_ of said subject over the year prior to T_0_. Each dot or triangle represents a subject (*n* = 46 in the PERT group, 40 in the control group)

HbA_1c_ decreased after the index date in both groups (Fig. 2a). Mean HbA1c decreased from 8.8 to 7.6% (73 to 60 mmol/mol) in the PERT group and from 8.2 to 7.6% (66 to 60 mmol/mol) in the control group (*p* = 0.0029 and *p* = 0.0038, respectively) (Fig. 2b). However, the intra-subject variability of HbA_1c_ was only of − 0.5% in the PERT group and of − 0.28% in the control group, and the difference between the groups was not significant (Fig. 2c).

#### BMI

**Fig. 3:**
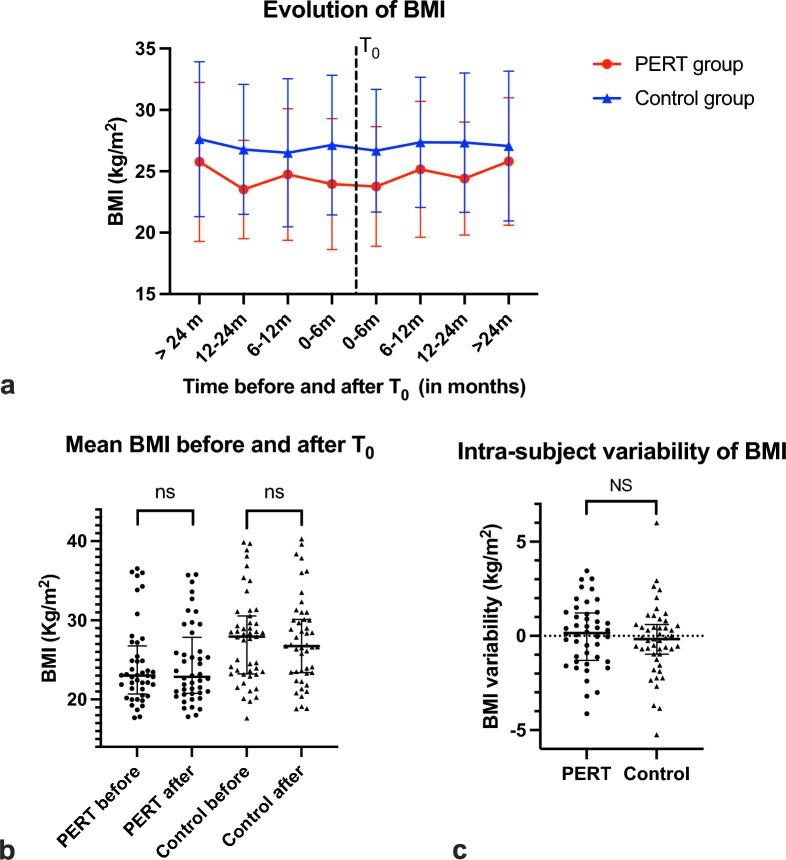
Evolution of BMI over the study period. **a** BMI (in kg/m^2^) at different time points before and after index date (T_0_). Red circles: PERT group. Blue triangles: control group. **b** Mean BMI (in kg/m^2^) over the 2 years prior and the 2 years following T_0_ in the PERT and control groups. Each dot or triangle represents a subject (*n* = 44 in the PERT group, 47 in the control group). **c** Intra-subject variability of BMI in the PERT and control groups. Intra-subject variability of BMI = mean BMI of subject over the 2 years following T_0_ – mean BMI of said subject over the 2 years prior to T_0_. Each dot or triangle represents a subject (*n* = 44 in the PERT group, 47 in the control group)

BMI was stable over the study period in both groups (Fig. 3a). Notably, it did not increase after PERT initiation in the PERT group (Fig. 3b and c). In subjects with CP, BMI tended to increase after PERT initiation, from 24.9 to 25.8 kg/m^2^, but the difference was not significant probably due to a lack of power.

#### Hyperglycemia after PERT initiation

In the PERT group, 9 subjects (19%: 2 subjects with T1D, 3 with pancreatogenic diabetes, 2 with MODY HNF-1B and one undetermined) had notable hyperglycemia in the months after PERT initiation. Among them, one reported only partial compliance to his diabetes medication. Six subjects had to increase their insulin dosage by a few units to improve glycemic control. One subject with MODY HNF-1B was prescribed repaglinide, which improved glycemic control. Another subject with MODY HNF-1B had to be initiated on insulin in a context of infection and acute renal failure, and could reverse back to oral medication afterwards. There was no report of ketoacidosis or hospitalization for hyperglycemia.

#### Adherence to PERT

Adherence to PERT can be rather difficult for subjects with EPI, because of the high number of capsules to take throughout the day. Three participants declared that they stopped taking PERT for several weeks or months, and 5 other participants had difficulty maintaining daily adherence to PERT. Among these 8 participants, 3 had gastro-intestinal disorders, which improved in only one subject after T_0_. 1 had relevant hypoglycemia, which did not improve after T_0_.

### Comparison between subjects with recommended dosage and underdosage of PERT

In the PERT group, 14 subjects were not prescribed the recommended dosage. They were prescribed a mean of 25,000 lipase units by meal. Thirty-four subjects were classified in the recommended dosage (RD) subgroup.

Relevant hypoglycemia symptoms improved in 55% (6 out of 11) of the RD subjects and 50% (2 out of 4) of the UD subjects (NS). Gastro-intestinal disorders improved in 87% (13 out of 15) of the RD subjects and 50% (2 out of 4) of the UD subjects (NS).

Mean HbA_1c_ decreased from 8.8 to 7.7% (73 to 61 mmol/mol) in the RD subgroup and from 8.9 to 7.6% (74 to 60 mmol/mol) in the UD subgroup (*p* = 0.04 and 0.02, respectively). Mean intra-subject variability of HbA_1c_ was of − 0.4 and − 0.9% in the RD and UD subgroups, respectively (NS). BMI was stable over the study period in both subgroups. Mean intra-subject variability of BMI was of − 0.12 and + 0.47 kg/m^2^ in the RD and UD subgroups, respectively (NS).

Out of the 9 subjects who had notable hyperglycemia after PERT initiation, 3 subjects were in the UD subgroup.

## Discussion

In this retrospective, monocentric study, we compared subjects with diabetes and EPI who were prescribed PERT for the first time, with control subjects who also presented with diabetes, had a FEC dosage and were not treated with PERT.

Frequent and or/severe hypoglycemia was a common occurrence in both groups, and it was more common in subjects treated with insulin. Over the study period, hypoglycemia symptoms improved in about half of the subjects, both in the PERT and the control groups. Thus, we reckon that in this study, PERT was probably not the main reason why hypoglycemia symptoms improved. PERT initiation and FEC dosage were mainly performed during hospitalization for educational purposes. The decrease in hypoglycemia frequency and severity might have resulted from improvement in diabetes management and in subjects’ autonomy. The decrease in HbA_1c_ in both groups over the study period was probably due to the same hospitalization effect. Our results are in accordance with Ewald et al.’s study [30], which did not show any difference in severe hypoglycemia rate after PERT initiation either. It must however be noted that in the present study relevant hypoglycemia symptoms improved in 80% of subjects with pancreatogenic diabetes. PERT might thus have a direct effect on hypoglycemia rate in subjects with pancreatogenic diabetes, but there were too few subjects to conclude definitively.

Conversely, the risk of severe hyperglycemia is often mentioned when PERT initiation is considered in subjects with diabetes [29]. We did not confirm this risk, as we did not report any keto-acidosis or hospitalization for hyperglycemia after PERT initiation. About one-fifth of the subjects had minor hyperglycemia after PERT initiation. Among these subjects, about 80% percent had to increase their insulin dosage by a few units, one subject had to be initiated on insulin in a context of infection and could reverse back to his usual treatment afterwards, and one was started on repaglinide. In the control group, one subject was initiated on insulin during the follow-up period. Moreover, HbA_1c_ globally decreased after PERT initiation. Hence, as was previously shown [27, 30], PERT initiation seems to be safe in subjects with diabetes. These subjects should be advised to check their blood glucose more closely in the weeks after PERT initiation in order to adapt their treatment if needed. On a more general note, side effects of PERT seem uncommon and mild in nature. Mild nausea and headaches have mostly been reported [9]. Aside from moderate hyperglycemia and occasional constipation, the subjects treated with PERT in the present study did not report any side effect of PERT.

Gastro-intestinal disorders were common in both groups. This was expected, since FEC dosage is often performed because of gastro-intestinal symptoms. Gastro-intestinal disorders improved more often after PERT initiation than in the control group. Gastro-intestinal disorders also tended to improve more often in patients with the recommended dosage of PERT than in patients who were under-dosed, even though the difference was non-significant, probably because of a lack of power in this subgroup analysis. Thus, we reckon that PERT has beneficial effects on gastro-intestinal disorders in subjects with diabetes.

BMI was globally stable over the period of study. PERT initiation has previously been associated to an increase in weight, mostly in subjects with pancreatogenic diabetes [33]. Our study does not confirm this, but BMI should be interpreted with caution in our population: GLP1 treatment was initiated at T_0_ in 5 subjects in the PERT group and 2 subjects in the control group. These subjects all lost or stabilized weight, as expected. Moreover, 3 subjects in the PERT group had pancreatic adenocarcinoma and lost weight progressively. Conversely, the BMI of subjects with CP and with T1D tended to increase after PERT initiation.

On another note, international guidelines recommend using 40,000–80,000 lipase units during main meals [31, 32]. These guidelines [34] were redacted for subjects with EPI and CP only, so there are no clear recommendations for subjects with more common types of diabetes and EPI. In the present study, a third of the subjects from the PERT group were not prescribed the recommended dosage. This is similar to what has been described elsewhere [35–37]. However, in the present study, HbA_1c_ evolution, BMI evolution, and hypoglycemia’s improvement rate were similar in subjects with recommended dosage or under-dosage of PERT. The rate of moderate hyperglycemia after PERT initiation was also similar between subgroups. Improvement in gastro-intestinal disorders seemed to be more common in subjects with higher dosages of PERT than in subjects with under-dosage, but the difference was not significant. This concurs with what has been described in the literature: PERT effects on short and medium-term symptoms do not seem to correlate with PERT dosage, except for gastro-intestinal disorders [35]. Adapting PERT dosage with the aim of relieving gastro-intestinal symptoms seems a possible way to find the right amount of PERT per meal, in order to improve quality of life [14]. Yet, some subjects remain symptomatic even with high dosages, implying that other causes than EPI might be responsible for diarrhea or abdominal pain and should be sought for [36].

Our study bears some limitations. This was a retrospective study and data were obtained from electronical medical records. Hence, the outcomes were only established on what was reported and written down in the records. When data were missing, we mentioned it in the corresponding table or figure. Moreover, continuous glucose monitoring (CGM) has been widely available only since 2017 in France, and until very recently was only offered to subjects living with T1D. Therefore, CGM was available only in a small subset of the participants and CGM data was too scarce to be analyzed.

This was a real-life study, which on the one hand, allowed us to study subjects with different types of diabetes, with different types of diabetes medication, with varying dosages of and varying adherence to PERT. On the other hand, all these variables make the interpretation of results more difficult and less reproducible. Moreover, because of the design of the study, we were not able to find out if PERT has an effect on osteoporosis rate or survival in subjects with diabetes.

Another limitation is the definition of EPI itself: in our Diabetology Department, it is common to prescribe PERT in subjects with evocative symptoms and a FEC < 100 µg/g. In CP, EPI is usually defined by a FEC < 200 µg/g but the FEC threshold for EPI definition is still in debate [38]. Some authors even reckon that FEC is not reliable to define EPI in subjects with T1D [39], but others showed that a FEC < 100 µg/g correlated well with steatorrhea in subjects with T1D and T2D [40]. When in doubt (namely if the first stool sample was watery or if pancreas imaging was normal), FEC dosage should be reproduced to confirm the EPI diagnosis.

## Conclusions

In a retrospective, monocentric, real-life study, we compared 48 subjects with EPI and any type of diabetes who were prescribed PERT for the first time with 48 control subjects with diabetes who were not prescribed PERT. PERT prescription was associated with an improvement in gastro-intestinal disorders, but not in relevant hypoglycemia rate, with the possible exception of subjects with chronic pancreatitis. HbA_1c_ and BMI evolution were similar between groups. PERT prescription was safe in subjects with diabetes, even though diabetes medication had to be mildly intensified in about one-fifth of the subjects after PERT initiation. PERT higher dosage tended to associate with stronger effects on gastro-intestinal disorders.

Hence, EPI should be sought for in subjects with diabetes and gastro-intestinal disorders, and PERT prescribed when appropriate. Prospective studies on PERT effects in subjects with diabetes would be useful to better understand its long-term effects in this population, and to confirm or infirm its beneficial effects on hypoglycemia rate and weight gain.

## Data Availability

The datasets generated during and/or analysed during the current study are available from the corresponding author on reasonable request.

## Abbreviations

PERT: Pancreatic enzyme replacement therapy
EPI: Exocrine pancreatic insufficiency
